# Anticipatory slow potentials before auditory feedback show posterior predominance but limited condition effects in speech-in-noise

**DOI:** 10.1016/j.ibneur.2026.05.013

**Published:** 2026-06-01

**Authors:** Kazuhiro Okamoto, Kengo Hoyano, Tomomi Nomura, Keisuke Irie, Naoya Obama, Narihiro Kodama, Yasutaka Kobayashi

**Affiliations:** a Department of Rehabilitation, Faculty of Health Science, Fukui Health Science University, Fukui, Japan; b Cognitive Motor Neuroscience, Department of Human Health Sciences, Graduate School of Medicine, Kyoto University, Kyoto, Japan; c Department of Speech and Language Therapy, Faculty of Health Rehabilitation, Kawasaki University of Medical Welfare, Kurashiki, Japan

**Keywords:** Speech-in-noise, Stimulus-preceding negativity, Contingent negative variation, EEG, Anticipatory neural activity, Linear mixed-effects model

## Abstract

It remains unclear whether noisy listening reliably modulates anticipatory EEG activity before feedback and whether such activity explains interindividual differences in speech-in-noise performance. We examined these questions in an independent cohort of young adults with normal hearing using a time-estimation task with auditory feedback presented in silence and in continuous multi-talker noise. Stimulus-preceding negativity (SPN) was quantified as the mean EEG amplitude in the −200–0 ms interval before feedback onset. The primary analysis used ROI × hemisphere linear mixed-effects models. A response-locked contingent negative variation (CNV) analysis was included as a control. Speech-in-noise performance was summarized descriptively across 0, −5, −10, and −15 dB signal-to-noise ratios, and brain–behavior associations were treated as exploratory. The primary SPN analysis showed a clear posterior predominance, with more negative amplitudes at parietal and occipital than at central and frontal regions. However, the main effect of listening condition was not significant, and neither hemisphere nor the condition × hemisphere interaction reached significance. The CNV control analysis likewise showed no reliable condition effect. Speech-in-noise performance declined monotonically as the signal-to-noise ratio decreased, indicating that the independent behavioral test captured the expected effect of increasing acoustic difficulty across SNR levels. Exploratory analyses did not reveal robust associations between speech-in-noise performance and SPN or CNV measures. These findings indicate that the task elicited anticipatory slow potentials with posterior predominance, but provided limited evidence for reliable modulation by background noise under the present task parameters and sample characteristics.

## Highlights

• Anticipatory EEG activity was tested during auditory feedback in silence and noise.
• The time-estimation task elicited SPN-like slow potentials before feedback onset.
• Slow potentials were strongest over posterior rather than central scalp regions.
• Background noise produced no reliable modulation of SPN or CNV amplitudes.
• EEG measures showed no robust association with speech-in-noise recognition.

## 1 Introduction

Everyday communication often requires speech decoding in noisy environments, such as conversing in a crowded restaurant or on a train platform surrounded by other speakers (Bottalico et al., 2022, Cherry and Wiley, 1967). In such settings, listeners must segregate auditory streams by aligning with the target speaker through a bottom-up analysis of acoustic features and top-down processing of linguistic cues, while concurrently suppressing irrelevant auditory input (Snyder and Alain, 2007, Tune et al., 2021). Top-down processing involves inferential mechanisms that resolve ambiguity in speech signals based on contextual information, including phonemes, syntax, and semantics (Obleser, 2014). Background noise introduces a substantial overlap in spectrotemporal information (McDermott, 2009) and bottom-up processing alone is insufficient, making top-down inference crucial for accurate perception (Zion-Golumbic et al., 2013, Leonard et al., 2016).

Predictive coding frameworks conceptualize speech perception as an inferential process in which the brain applies Bayesian principles to anticipate upcoming speech input (Friston, 2010, Harding et al., 2024). Prediction errors occur when sensory input deviates from expectations, propagate across cortical hierarchies to update internal models, and facilitate adaptive comprehension. Recent studies have demonstrated that the electrophysiological indices of speech prediction correlate with speech perception performance in the presence of noise (Okamoto et al., 2024). Other studies have reported that cortical speech tracking is related to individual prediction tendency and interacts with background noise during speech processing (Schubert et al., 2023). Collectively, these findings indicate that listeners differ in how strongly they engage in top-down processes during speech perception in noisy environments. Whether background noise alters anticipatory neural activity before feedback, and whether such activity is related to individual variability in speech perception under masking are more conservative questions.

Predictive dynamics can be measured using EEG in an S1–S2 paradigm, in which a cue stimulus (S1) precedes a target stimulus (S2). Tasks requiring a motor response to S2 typically elicit a gradual negative-going potential and a contingent negative variation (CNV) during the S1–S2 interval (Kononowicz and Penney, 2016, Ruiz-Martínez et al., 2022). A similar slow negative shift, known as the stimulus-preceding negativity (SPN), can emerge before an expected event, even when no overt response is required for the upcoming stimulus, and is generally interpreted as reflecting anticipatory processing. Because SPNs develop before an expected event, it has often been discussed in relation to both predictive and anticipatory processes (Chwilla and Brunia, 1991). Some theoretical and simulation studies have discussed the role of SPN within predictive processing frameworks (Bennett et al., 2015, Gómez et al., 2019), while contemporary accounts often regard it as a domain-general anticipatory signal, rather than a speech-specific marker (Ono et al., 2021). As such, larger SPN amplitudes may reflect stronger anticipatory engagement before an expected event. Simultaneously, the anticipated slow potentials may also be influenced by factors such as attentional allocation, task engagement, and preparatory processes (Kotani et al., 2025). Accordingly, in the present study, we treated the SPN as an index of anticipatory neural activity, rather than a process-pure marker of prediction. Our prior study observed larger SPN amplitudes under noise (Okamoto et al., 2024), but did not examine whether this modulation could explain individual performance variability. Therefore, the present study used a fully independent cohort to reexamine this effect using a revised and more conservative analytical framework. Specifically, we focused on three questions: (i) whether background noise modulates anticipatory SPN amplitude, (ii) how are any condition effects distributed across the scalp, and (iii) whether anticipatory EEG activity is associated with any individual differences in speech-in-noise recognition across different signal-to-noise ratio (SNR) levels. Rather than assuming that the SPN reflects prediction alone, we treated it as an anticipatory signal whose functional significance may include prediction, attentional allocation, listening effort, and other preparatory processes.

Herein, we tested the following two hypotheses: Hypothesis 1 proposed that background noise amplifies SPN, which is consistent with enhanced anticipatory neural engagement when sensory evidence is degraded. Hypothesis 2 posited that a larger SPN alone does not guarantee better recognition; the SPN reflects preparatory readiness, whereas recognition accuracy depends on the fidelity of the sensory input, allocation of attentional resources, and accessibility of linguistic representations. If both hypotheses hold true, the SPN could be interpreted as a neural correlate of anticipatory readiness during noisy listening, rather than as a sufficient or process-specific determinant of successful speech-in-noise perception.

## 2 Materials and methods

### 2.1 Participants

The present study used a fully independent cohort in which no participants overlapped with the sample reported by Okamoto et al. (2024). This study was designed as an independent extension of our prior report, placing a stronger focus on the spatial distribution of the condition effect and robustness of the EEG results under a revised non-interpolated primary analysis. To minimize the confounding effects of age-related auditory variations, recruitment was restricted to individuals aged 18–23 years. The cohort included 26 participants (13 females and 13 males), all of whom provided written informed consent before enrollment. Sex was defined as the sex assigned at birth based on external anatomical characteristics as self-reported by the participants. All the participants received monetary compensation for their involvement. Standard audiometric screening was conducted to verify normal bilateral hearing, defined as ≤ 20 dB HL pure-tone averages across 125–8000 Hz (American Speech-Language-Hearing Association, 1990). Sustained auditory attention was evaluated using an auditory Continuous Performance Test (a-CPT; Fujimoto et al., 2019), with all participants demonstrating performance within the normal range. Handedness was assessed using the Japanese version of the simplified Edinburgh Handedness Inventory (Oldfield, 1971), and all participants were classified as right-handed. The participants also self-reported having normal or corrected-to-normal vision, no history of psychiatric or neurological disorders, and native proficiency in Japanese. Datasets with fewer than 15 valid trials were excluded from the EEG analyses. In addition, as the primary analyses were based on non-interpolated EEG data, according to pre-specified quality control criteria, any runs with extensive channel loss were excluded. Specifically, runs with 10 or more bad channels were excluded from the primary analyses, and participants whose available runs had seven or more bad channels were excluded at the participant level. The number and proportion of bad channels and the resulting exclusions are reported in the Results section.

This study was approved by the Research Ethics Committee of Fukui University of Health Sciences (Approval No. 2022–14) and was conducted in accordance with the Declaration of Helsinki. The methods and results were reported following the recommendations of the OHBM Committee on Best Practices in Data Analysis and Sharing for M/EEG (COBIDAS-MEEG) (Pernet et al., 2020; COBIDAS-MEEG White Paper, OSF, accessed Sep 17, 2025).

### 2.2 Apparatus

The auditory setup consisted of three 8330AP loudspeakers (GENELEC, Tokyo, Japan) positioned 1 m from the participant: one directly in front at 0°, and two angled at ±45°. The central loudspeaker (0°) delivered the target stimuli, whereas the lateral loudspeakers (+45° and −45°) broadcast background noise. The sound pressure levels were calibrated at the ear position (1 m, 0°) using a class−1 sound level meter (NL−20; RION, Tokyo, Japan) equipped with a ½-inch free-field microphone. Visual instructions and task cues were presented on a 14-inch liquid LCD monitor placed 1 m in front of each participant. Stimulus presentation was controlled using PsychoPy v2022.1.4 (Peirce, 2007), and all sessions were conducted inside a sound-insulated chamber. The ambient background noise in the booth was 45.3 dB(A).

### 2.3 Stimuli

Auditory feedback and multi-talker noise were recorded using a C480 B condenser microphone (AKG, Vienna, Austria) connected to a DR−680 MK II recorder (TASCAM, Tokyo, Japan). The audio was digitized at a resolution of 48 kHz/24-bit. Recordings from each speaker were digitally root mean square (RMS)-normalized, equally weighted, and summed to generate multitalker noise. The noise was designed to emulate a realistic background environment similar to a crowded restaurant, comprising speech from 10 speakers (five males, five females; all in their twenties) reading aloud for 10 min.

To spatially distribute the noise, we introduced interchannel time differences between the two loudspeakers to create multiple perceived sound images. In the present study, this procedure was used to generate the perception of ten distributed source directions, thereby producing a spatially diffuse noise field. For an audio signal, f(t), a delayed version, f(t−∆t), was presented from one loudspeaker, while the original was presented distinct from the other. The ∆t values were adjusted to simulate perceived azimuthal angles of ±82.6° (0.63 ms), ±62.3° (0.57 ms), ±48.1° (0.48 ms), ±32.1° (0.34 ms), and ±16.5° (0.18 ms). Here, “sound image” refers to the perceived auditory location, rather than the physical loudspeaker position (cf. Harima et al., 1997).

The target stimulus was presented from a front loudspeaker at 65 dB SPL. Under the noisy condition, the two lateral loudspeakers were adjusted such that their combined equivalent continuous noise level (Leq) at that position was 65 dB SPL, corresponding to an SNR of 0 dB relative to the target. Each lateral loudspeaker produced an SPL of approximately 62 dB when measured individually. In the noisy condition, the masker was presented continuously from the two lateral loudspeakers throughout each block, while no masker was presented in the silent condition. Therefore, the total presentation levels were 65 dB SPL and 68 dB SPL in silence and noise, respectively.

### 2.4 Quantifying anticipatory neural activity before auditory feedback

**Fig. 1:**
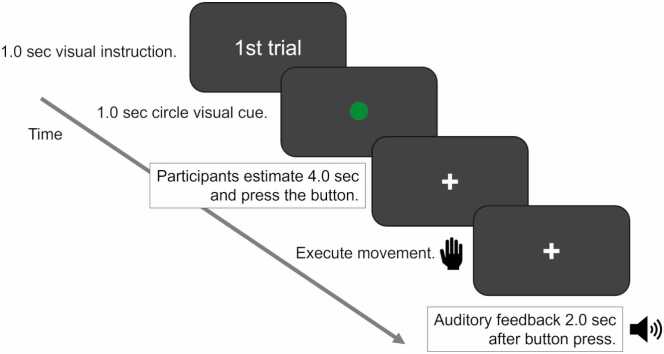
Overview of the experimental design. The participants were instructed to press a button when they subjectively estimated that 4 s had elapsed. Two seconds after the button was pressed, auditory feedback indicating the accuracy of the estimation was presented. Stimulus-preceding negativity (SPN) was defined as the mean EEG activity in the −200–0 ms window preceding feedback onset.

To quantify the anticipatory neural activity before auditory feedback, we used a time-estimation task that has previously been shown to elicit stimulus-preceding negativity (SPN) prior to feedback onset (Ohgami et al., 2021). A green circle appeared for 1 s and then switched to a fixation cross, marking the start of the estimation interval. The participants pressed a response key when they judged that 4 s had elapsed. Two seconds after this press, a front-center loudspeaker presented auditory feedback (S2), regardless of accuracy. Feedback consisted of a recorded Japanese word indicating correctness (“atari”) or incorrectness (“hazure”). Feedback was defined as correct (“atari”) if the participant pressed the button within ±0.5 s of the 4 s target interval; responses before 3.5 s or after 4.5 s were categorized as incorrect (“hazure”) (Fig. 1). Responses were made with the right index finger and the participants were instructed to maintain identical movements across the conditions. They were also instructed not to count aloud or subvocally during estimation.

The task included two listening conditions: a quiet condition (*in silence*) and a noisy condition (*in noise*). Under noisy conditions, the multitalker noise was presented continuously throughout the block from two lateral loudspeakers at an overall SNR of 0 dB relative to the target feedback word. Because the stimulus-onset asynchrony between response and feedback was fixed, both conditions involved a temporal expectancy regarding feedback onset. Simultaneously, spoken feedback encouraged the participants to anticipate the likely feedback content based on their performance. However, the present design was not intended to isolate predictions from other processes that could contribute to anticipatory slow potentials, including attentional allocation, task engagement, listening effort, and preparation for upcoming stimulus processing. Accordingly, any condition difference in the SPN was interpreted as a difference in the anticipatory neural activity under different auditory contexts, rather than as evidence for prediction alone.

Participants visited the laboratory on two separate days. During their first visit, they received instructions, and completed at least 12 practice trials. After training, they completed an auditory imagery questionnaire and a speech-in-noise perception task. The experimental EEG session was conducted on the second day and comprised 120 trials arranged in four 30-trial blocks. The two listening conditions alternated across blocks. For each participant, the starting condition was randomized, resulting in either a silence–noise–silence–noise sequence or a noise–silence–noise–silence sequence. After each block, the participants received percentage feedback on their time-estimation performance and took a three-minute break.

### 2.5 Speech perception test in noise

Speech perception in noise was assessed using a method developed for native Japanese speakers (Fujimoto et al., 2019). The stimulus set comprised two-syllable Japanese words balanced for lexical frequency and phonotactic constraints, with no repetitions within the participants. To ensure consistency in the auditory presentation, the same loudspeaker configuration employed in the time-estimation task was used. The task consisted of four blocks, each corresponding to a different SNR: 0 dB, −5 dB, −10 dB, and −15 dB. In each block, multi-talker noise was presented from the left and right speakers to establish a specified SNR level. A total of 24 trials were conducted (six per SNR). During the trials, 24 words were randomly presented to a front-facing speaker, and the number of correctly identified words was recorded as an accuracy score. Performance in this task was summarized descriptively at each SNR, and used for exploratory brain-behavior analyses.

### 2.6 EEG recording and preprocessing

EEG signals were acquired using an NVX24 amplifier and MCScap Ag/AgCl 24-electrode cap (Medical Computer Systems, Zelenograd, Moscow). Signals were digitized at a 24-bit resolution, with a sixth-order delta-sigma modulator operating at 64 × oversampling, employing one converter per channel. The dynamic input range of EEG channels was 400 mV. The amplifier applied an analog anti-aliasing low-pass filter whose −3 dB cutoff frequency varied according to the sampling rate. Data were sampled at 500 Hz and recorded with an online 0.5-Hz high-pass, 70-Hz low-pass, and 60-Hz main notch filter. Electrodes were placed according to the international 10–20 system, and EEG data were recorded from 19 scalp sites: Fp1/2, F3/4, C3/4, P3/4, O1/2, F7/8, T3/4, and T5/6, and midline positions Fz, Cz, and Pz. Grounding was established at the frontal pole (Fpz), with linked earlobes (A1, A2) serving as references, and electrode impedance was maintained below 10 kΩ throughout the recording.

EEG pre-processing was conducted using EEGLAB (version 2024.0; Delorme and Makeig, 2004). Continuous data were low-pass filtered at 30 Hz for the ERP analyses. Bad channels were identified using the PREP pipeline (Bigdely-Shamlo et al., 2015) and excluded from independent component analysis (ICA) using the extended Infomax algorithm (Lee et al., 1999). The ICA was trained on a copy of the good-channel data, high-pass filtered at 1 Hz, and the resulting ICA weights were transferred to the corresponding low-pass dataset, thereby preserving the low-frequency activity. Independent components were screened using ICLabel as an auxiliary classifier (Pion-Tonachini et al., 2019) and final rejection decisions were made by visual inspection of scalp maps, activation time courses, and power spectra. Following ICA correction, the SPN- and CNV-related epochs were extracted, automatically screened using a ± 100 µV threshold on scalp EEG channels only, and finally manually reviewed (Kotani et al., 2025). For the primary analyses, non-interpolated data were used. Interpolated datasets were retained for supplementary and descriptive purposes.

### 2.7 EEG analysis

For SPN analysis, data were segmented from −2.0 to 0.3 s relative to feedback onset (S2). Baseline correction used an interval from −1.5 to −1.2 s, following previous SPN studies that used time-estimation tasks (Bhangal et al., 2021, Ohgami et al., 2014, Ohgami et al., 2023). SPN amplitude was defined as the mean EEG amplitude in the −200–0 ms interval, immediately preceding the feedback onset (Brunia and Damen, 1988, Kotani and Aihara, 1999, Kotani et al., 2015, Ohgami et al., 2021, Raiha et al., 2020). Trial-wise SPN values were retained at the electrode level, and were subsequently summarized within the ROI/hemisphere cells for primary analyses.

To address the possibility that any SPN effect may reflect a more general preparatory activity than feedback-related anticipation, we included a response-locked CNV control analysis. If the noise effect was observed for both the SPN and CNV, it would be more consistent with a broader change in anticipatory or preparatory states under noisy listening. For this analysis, epochs were extracted from −1.2 to 0.2 s relative to the response, and the baseline correction used the −1.2 to −1.0 s interval relative to the response. CNV amplitude was quantified in the −0.5 to −0.1 s pre-response interval, chosen to capture the late preparatory negativity, while reducing contamination from activity immediately surrounding motor execution. This choice was guided by the distinction between the early and late CNV phases, with the late phase being more closely related to response preparation (Brunia and van Boxtel, 2001).

### 2.8 Statistical analysis

As interpolation should be used cautiously in a 19-channel montage, the primary analysis was based on the non-interpolated data. Runs with ten or more bad channels were excluded from the primary analysis. In addition, participants whose runs consistently showed extensive channel loss (defined as seven or more bad channels across all runs) were excluded from the study. The ROI averages were computed only when at least 50% of electrodes within a given ROI/hemisphere cell were available.

We did not conduct a formal a priori power analysis for the linear mixed-effects models (LMMs) because closed-form solutions depend on unknown variance components, including subject-level random-effects variances and within-subject correlations. Rather than reporting retrospective power, we focused on estimation. Model results are therefore reported as point estimates with 95% confidence intervals, and multiplicity was controlled where applicable.

The primary analysis tested the effect of background noise on anticipatory EEG activity using ROI × hemisphere LMMs that accounted for repeated observations within participants. For the primary SPN analysis, the trial-level mean SPN amplitude within each ROI × hemisphere cell was used as the dependent variable. Only participants contributing observations in both listening conditions were included, such that condition contrasts were evaluated within a common population. Fixed effects were sum-coded (Condition: *in noise* = −0.5, *in silence* = +0.5; Hemisphere: right = −0.5, left = +0.5) to facilitate interpretation of the intercept. Fixed effects included Condition, ROI, Hemisphere, and the Condition × Hemisphere interaction. The central ROI was included in all ROI-based models and served as the reference level for the ROI factor. Therefore, ROI coefficients represent differences from the central ROI. To evaluate whether condition effects varied across scalp regions, we also fitted supplementary ROI-interaction models that included Condition × ROI terms for both SPN and CNV. These models used the same dependent variables, condition coding, and random-effects strategy as the primary ROI × hemisphere models. Random effects included participant-specific intercepts and by-condition slopes; when models did not converge, the random-effects structure was simplified to intercept-only. Models were estimated using restricted maximum likelihood. We report fixed-effect estimates (*b*), standard errors (SE), *z* values, *p* values, and 95% confidence intervals (CI). As a control analysis, we fitted an analogous ROI × hemisphere LMM to the response-locked CNV data. This analysis was intended to determine whether any condition-related modulation of anticipatory activity was specific to the interval preceding auditory feedback or generalized to a broader response-locked preparatory negativity. The CNV model used the same fixed- and random-effects structures as the primary SPN model. To quantify the extent to which the data supported reduced models without condition-related terms, we conducted supplementary Bayes factor analyses based on the Bayesian information criterion (BIC). These analyses were performed for both SPN and CNV models. For each comparison, the reduced and full models were fitted using maximum likelihood, and BIC differences were converted to approximate Bayes factors. For both SPN and CNV, these comparisons tested the Condition main effect, all condition-related terms in the primary ROI × hemisphere model, and the additional contribution of Condition × ROI terms beyond the primary model. These analyses were treated as sensitivity analyses rather than primary inferential tests because they depend on model specification and provide an approximation rather than a fully Bayesian mixed-effects analysis.

To visualize the spatial distribution of the SPN pattern, we computed sensor-space topographies for the in-silence condition, the in-noise condition, and the *in noise* minus *in silence* difference. In addition, as an exploratory spatial analysis, a two-sided one-sample cluster-based permutation procedure was applied to participant-level noise-minus-silence difference maps using electrode adjacency defined from standard 10–20 coordinates and 5000 randomizations. The same procedure was also applied to the response-locked CNV difference maps as a control analysis. These cluster-based analyses were used to evaluate whether the condition contrast showed spatially clustered evidence across electrodes, but they were not used to define follow-up ROIs or cluster-averaged confirmatory tests.

Several supplementary and exploratory analyses were conducted. First, sex-adjusted versions of the SPN and CNV ROI × hemisphere LMMs were fitted as supplementary robustness checks. Second, separate electrode-wise LMMs were fit to test the main effect of the condition at each electrode, with multiplicity controlled using the Benjamini–Hochberg false discovery rate (FDR) procedure. Third, the speech-in-noise performance was summarized descriptively at each SNR level, while participant-level associations between speech-in-noise performance and EEG measures were examined only exploratorily because the behavioral metric offered limited interindividual variability in this young normal-hearing sample. Accordingly, these brain-behavior analyses were not treated as primary inferential tests.

All analyses were conducted in Python 3.11 (Python Software Foundation, 2022). LMMs were fitted using statsmodels, sensor-space cluster-based permutation procedures were implemented in MNE-Python, and ancillary computations were performed using NumPy and SciPy.

## 3 Results

### 3.1 Sample retention and EEG quality control

Of the 26 enrolled participants, six were excluded from the primary EEG analysis. Four participants were excluded because they did not meet the minimum valid-trial criterion after artifact rejection, defined as fewer than 15 valid trials in at least one required condition or analysis cell. One participant was excluded because of a procedural deviation during EEG recording related to improper electrode placement. One additional participant was excluded at the participant level because all available recordings showed seven or more bad channels. After these exclusions, 74 runs from 20 participants were retained for the primary EEG analyses. Across the retained EEG runs, the mean number of bad channels was 4.10 ± 1.89, corresponding to 21.6% ± 9.9% of the 19-channel montage (range = 1–8 channels). None of the retained runs met the run-level exclusion criterion of 10 or more bad channels. The participant characteristics of the final analytical sample are summarized in Table 1.

**Table 1:** Participant characteristics and speech-in-noise performance.

Participant characteristics		N = 20
Sex assigned at birth, n (%)		Male: 9 (45%), Female: 11 (55%)
Age (years)		20.3 ± 1.35
Hearing Threshold (dBHL)	Right Ear	5.90 ± 4.03
	Left Ear	5.02 ± 3.60
a-CPT score (%)		99.5 ± 0.02
**Speech-in-noise performance**	SNR (dB)	
Correct responses (out of 6)	0	5.55 ± 0.51
	−5	4.50 ± 1.19
	−10	1.20 ± 0.95
	−15	0.80 ± 0.77

**Abbreviations:** SD, standard deviation; dB HL, decibel hearing level; a-CPT, auditory continuous performance test. **Note:** Data are mean ± standard deviation unless indicated. Speech-in-noise values are shown for the 20 participants included in the primary EEG analyses. Correct responses are reported out of 6 trials at each SNR. Corresponding mean accuracy values were 0.92 ± 0.09, 0.75 ± 0.20, 0.20 ± 0.16, and 0.13 ± 0.13 at 0, −5, −10, and −15 dB SNR, respectively.

Across valid trial records included in the EEG dataset, “atari” feedback occurred on 526 of 835 records *in noise* condition and on 515 of 875 records *in silence* condition; the remaining records were followed by “hazure” feedback.

### 3.2 Primary SPN ROI × hemisphere analysis

**Fig. 2:**
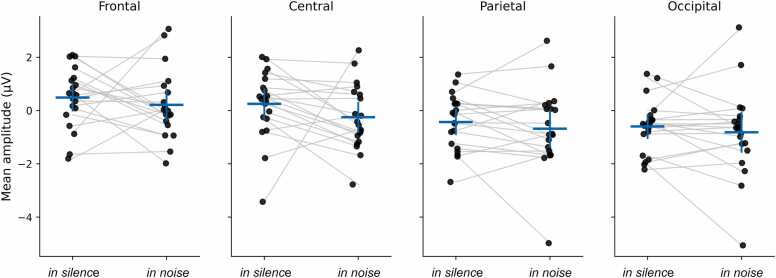
Mean SPN amplitude by ROI and listening condition. The panels show the participant-level mean SPN amplitudes for the Frontal, Central, Parietal, and Occipital ROIs in the *in silence* and *in noise* conditions. Each point represents one participant, while thin gray lines connect the within-participant values across the conditions. Blue horizontal bars indicate the mean, while vertical whiskers indicate the 95% confidence interval. Larger negative values indicate larger SPN amplitudes.

The primary ROI × Hemisphere LMM based on the non-interpolated SPN data showed a clear posterior predominance (Table 2). Relative to the central ROI, SPN amplitudes were less negative at the frontal sites (*b* = 0.333, SE = 0.118, z = 2.82, *p* = 0.005), but more negative at the occipital (*b* = −0.730, SE = 0.129, z = −5.66, *p* < 0.001) and parietal sites (*b* = −0.600, SE = 0.116, z = −5.18, *p* < 0.001). In contrast, the main effects of the condition (*b* = 0.318, SE = 0.362, z = 0.88, *p* = 0.380) and hemisphere (*b* = 0.046, SE = 0.100, z = 0.46, *p* = 0.647) were both insignificant. The Condition × hemisphere interaction was also not significant (*b* = 0.139, SE = 0.199, z = 0.70, *p* = 0.484). Thus, the primary analysis indicated a robust posterior distribution of anticipatory negativity, but did not provide evidence that the SPN amplitude differed reliably between the *in noise* and *in silence* conditions (Fig. 2). Supplementary electrode-wise analyses of the SPN are reported in Table S1. A supplementary sensitivity analysis using interpolated data yielded the same overall pattern of SPN results, including posterior predominance and the absence of a reliable condition effect (Table S2). To further address whether condition effects differed across scalp regions, we fitted an additional ROI-interaction model including Condition × ROI terms. The central ROI was included in this model and served as the reference level. In this model, the condition effect at the central ROI was not significant (*b* = 0.512 µV, SE = 0.393, *z* = 1.30, *p* = 0.193). None of the Condition × ROI terms reached significance: frontal ROI, *b* = −0.225 µV, SE = 0.236, *z* = −0.95, *p* = 0.341; occipital ROI, *b* = −0.297 µV, SE = 0.258, *z* = −1.15, *p* = 0.250; and parietal ROI, *b* = −0.249 µV, SE = 0.232, *z* = −1.07, *p* = 0.283. Supplementary BIC-based Bayes factor analyses were consistent with the frequentist results. For SPN, the reduced model without the Condition term was favored over the model including the Condition main effect (BF01 = 134.9). The reduced model without condition-related terms was also favored over the primary model including Condition and Condition × Hemisphere terms (BF01 = 15,571). In addition, the model without Condition × ROI terms was favored over the ROI-interaction model (BF01 = 1.42 × 10^6^). These results indicate that the present data provided limited support for adding condition-related terms to the SPN model. Table 3

**Table 2:** Primary ROI × Hemisphere linear mixed-effects model for non-interpolated SPN amplitude.

	*b* (µV)	SE	*z*	*p*	95% CI
ROI: Frontal	0.333	0.118	2.823	0.005	[0.101, 0.564]
ROI: Occipital	−0.730	0.129	−5.662	< 0.001	[−0.983, −0.477]
ROI: Parietal	−0.600	0.116	−5.181	< 0.001	[−0.827, −0.373]
Condition	0.318	0.362	0.878	0.380	[−0.391, 1.027]
Hemisphere	0.046	0.100	0.459	0.647	[−0.149, 0.241]
Condition × Hemisphere	0.139	0.199	0.700	0.484	[−0.250, 0.529]

**Abbreviations:***b* = fixed-effect coefficient; SE = standard error; *z* = Wald z statistic; *p* = two-sided p-value; 95% CI = 95% confidence interval; SPN = stimulus-preceding negativity; ROI = region of interest; µV = microvolts.

**Note:** The central ROI served as the reference level for the ROI factor and was included in the model.

**Table 3:** ROI × Hemisphere linear mixed-effects model for non-interpolated response-locked CNV amplitude.

	*b* (µV)	SE	*z*	*p*	95% CI
ROI: Frontal	0.318	0.114	2.780	0.005	[0.093, 0.542]
ROI: Occipital	−0.006	0.125	−0.050	0.960	[−0.251, 0.239]
ROI: Parietal	−0.134	0.112	−1.198	0.231	[−0.354, 0.085]
Condition	−0.057	0.361	−0.158	0.874	[−0.764, 0.650]
Hemisphere	0.111	0.096	1.154	0.249	[−0.078, 0.300]
Condition × Hemisphere	0.033	0.193	0.170	0.865	[−0.345, 0.410]

**Abbreviations:***b* = fixed-effect coefficient; SE = standard error; *z* = Wald z statistic; *p* = two-sided p-value; 95% CI = 95% confidence interval; CNV = contingent negative variation; ROI = region of interest; µV = microvolts.

**Note:** The central ROI served as the reference level for the ROI factor and was included in the model.

### 3.3 Descriptive sensor-space localization of the SPN condition effect

**Fig. 3:**
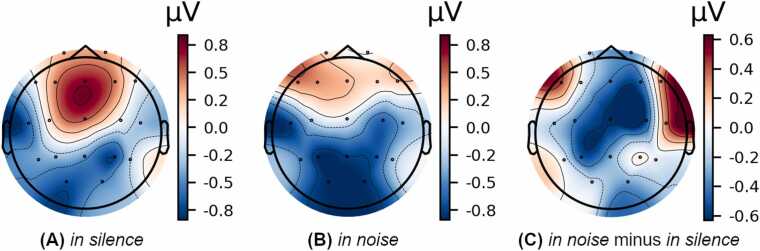
Descriptive SPN topographies for the silence and noise conditions, and the noise-minus-silence difference. (A) Topographical map of the mean SPN amplitude in the *in silence* condition, computed in the −200–0 ms interval prior to feedback onset. (B) Topographical map of the mean SPN amplitude in the *in noise* condition, computed in the same interval. (C) Topographical map of the difference wave (*in noise* minus *in silence*) for the mean SPN amplitude in the −200–0 ms interval prior to feedback onset. More negative values indicate larger SPN amplitudes. The difference map is shown for descriptive localization only, and was not used to define inferential follow-up analyses. The *in silence* and *in noise* maps were plotted using the same color scale. The difference map used a separate symmetric color scale to visualize the smaller *in noise* minus *in silence* difference. Cluster-based permutation analysis did not identify any suprathreshold spatial cluster for the *in noise* minus *in silence* contrast.

**Fig. 4:**
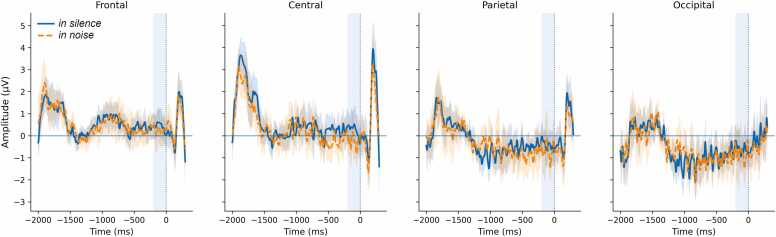
Descriptive SPN waveforms by ROI *in silence* and *in noise* conditions. Grand-average SPN waveforms are shown separately for the frontal, central, parietal, and occipital ROIs *in silence* and *in noise* conditions. The shaded region indicates the SPN measurement window (−200–0 ms relative to feedback onset), and the vertical dashed line marks feedback onset (0 ms). Shaded bands indicate variability across participants. More negative values indicate larger anticipatory negativity.

To visualize the spatial distribution of anticipatory activity, we computed sensor-space topographies for the SPN in silence, the SPN in noise, and the *in noise* minus *in silence* difference (Fig. 3). The *in silence* and *in noise* maps were plotted using the same color scale to allow direct visual comparison between the two listening conditions. Both condition-specific maps showed a broadly posterior negative distribution. The difference map showed a modest central tendency in the noise-minus-silence contrast, although no suprathreshold spatial cluster was identified in the exploratory cluster-based permutation analysis. In addition, descriptive ROI-wise waveform plots (Fig. 4) showed a more negative pre-feedback shift at posterior than at frontal and central regions, but only modest differences between the in-silence and in-noise conditions. The waveform morphology did not show a strongly monotonic ramping pattern toward feedback onset. The exploratory cluster-based permutation analysis of the participant-level noise-minus-silence SPN maps did not identify any suprathreshold spatial cluster. The corresponding CNV analysis likewise did not identify any suprathreshold cluster. Because these analyses were applied to the non-interpolated complete-case electrode set, they were treated as supplementary spatial checks rather than as the primary basis for inference.

### 3.4 Response-locked CNV control analysis

To assess whether any condition effect was generalized to the response-related anticipatory activity, we fitted the same ROI × Hemisphere LMM structure to the non-interpolated response-locked CNV data. As in the SPN analysis, the main effect of Condition was not significant (*b* = −0.057, SE = 0.361, z = −0.16, *p* = 0.874), while neither the main effect of Hemisphere (*b* = 0.111, SE = 0.096, z = 1.15, *p* = 0.249) nor the Condition × Hemisphere interaction (*b* = 0.033, SE = 0.193, z = 0.17, *p* = 0.865) reached significance. The only significant ROI effect was that the frontal amplitudes were less negative than the central amplitudes (*b* = 0.318, SE = 0.114, z = 2.78, *p* = 0.005), while the occipital and parietal amplitudes did not differ significantly from the central values. Thus, the control analysis did not provide any evidence for reliable modulation of the anticipatory activity by listening conditions. Electrode-wise supplementary CNV results are reported in Table S3. A corresponding interpolated sensitivity analysis for the response-locked CNV likewise did not reveal any reliable condition effect (Table S2). The supplementary CNV ROI-interaction model also did not provide evidence for region-specific condition effects. The condition effect at the central ROI was not significant (*b* = −0.155 µV, SE = 0.389, *z* = −0.40, *p* = 0.691), and none of the Condition × ROI terms reached significance (all *p* ≥ 0.140). The supplementary BIC-based Bayes factor analyses showed a similar pattern for CNV. The reduced model without the Condition term was favored over the model including the Condition main effect (BF01 = 148.4), and reduced models were also favored over models including all condition-related terms in the primary model (BF01 = 21786) or additional Condition × ROI terms (BF01 = 25669). Thus, the CNV analysis also provided limited support for condition-related modulation. These Bayes factor results are summarized in Table S6.

### 3.5 Speech-in-noise performance and exploratory brain–behavior analyses

In the independent speech-in-noise test, recognition accuracy decreased as the SNR became poorer. The mean accuracy was 0.92 ± 0.09 at 0 dB, 0.75 ± 0.20 at −5 dB, 0.20 ± 0.16 at −10 dB, and 0.13 ± 0.13 at −15 dB (Table 1). Thus, the speech-in-noise test captured the expected effect of increasing acoustic difficulty across SNR levels. However, the EEG task itself contrasted the in-silence condition with a 0 dB SNR noise condition, and performance at 0 dB was high in this young normal-hearing sample. Because of this restricted variability, the associations between speech-in-noise performance and anticipatory EEG measurements were treated as exploratory. Exploratory participant-level analyses did not reveal any robust associations between speech-in-noise accuracy and SPN or CNV. No exploratory associations reached conventional significance across the speech performance indices tested. Detailed exploratory participant-level associations between speech-in-noise performance and SPN/CNV measures are reported in Table S4.

## 4 Discussion

Overall, the present study re-examined the anticipatory neural activity before auditory feedback under quiet and noisy listening conditions using a revised and more conservative analytical framework centered on the ROI × hemisphere mixed-effects models. The principal finding was a clear posterior predominance of anticipatory negativity, with more negative amplitudes in the parietal and occipital regions than in the central or frontal regions. However, contrary to our initial expectations, the primary analysis did not reveal any reliable main effect of listening condition on SPN. Similarly, the parallel response-locked CNV control analysis showed no significant condition effects. In the independent speech-in-noise test, recognition accuracy decreased as SNR became poorer, indicating that the behavioral test captured the expected effect of increasing acoustic difficulty. However, the EEG task contrasted silence with 0 dB SNR noise, and recognition accuracy at 0 dB was close to ceiling in this young normal-hearing sample. The exploratory analyses did not identify any robust associations between speech-in-noise performance and either SPN or CNV measures. Taken together, these results indicate that the present task elicited anticipatory slow potentials, with a clear posterior distribution but did not provide strong evidence that background noise reliably enhanced these signals at the group level in this independent cohort.

The clearest result in the EEG data concerned the spatial distribution, rather than condition-related modulation. In the primary SPN analysis, the occipital and parietal regions showed more negative amplitudes than the central sites, whereas the frontal regions showed fewer negative amplitudes. This posterior predominance is consistent with the view that the task elicits a broadly distributed anticipatory slow potential rather than a focal effect confined to a small number of electrodes (Catena et al., 2012). The descriptive topographies further suggested broadly similar posterior negative configurations in both of the listening conditions. As the listening-condition effect itself was not reliable in the primary inferential model, we interpreted these topographies descriptively, and avoided strong anatomical claims regarding the condition-specific engagement of distinct hemispheric or frontoparietal control systems.

The descriptive waveform plots further clarified the morphology of the anticipatory activity. Although the posterior ROIs showed a more negative pre-feedback shift than the frontal and central ROIs, the waveforms did not show a strongly canonical ramping pattern toward feedback onset. In addition, the in-silence and in-noise waveforms remained relatively close across ROIs, which is consistent with the absence of a reliable condition effect in the primary LMM. Thus, the present task appeared to elicit a recognizable anticipatory slow potential with posterior predominance, but its temporal profile did not fully resemble the strongly ramping SPN sometimes described in simpler feedback-anticipation paradigms.

These findings suggest caution in attributing the anticipatory slow potentials in the present task to background noise-driven enhancement of predictive processing. In principle, a noisy listening context may increase the reliance on internal models, contextual constraints, or expectancy-related processes. (Obleser and Kotz, 2010, Van Os et al., 2022, Sharma et al., 2016) At the same time, noisy listening may also increase attentional allocation, task engagement, and listening effort (Peelle, 2018). The present data do not support the claim that background noise reliably amplifies anticipatory activity prior to feedback. Instead, the results were consistent with the interpretation that anticipatory neural activity was elicited in both of the listening contexts, whereas the degree of modulation by background noise was modest and statistically unreliable in the current sample. Although the descriptive topographies and electrode-wise estimates suggested a modest tendency toward stronger central negativity *in noise* condition, this pattern was not statistically reliable. The largest uncorrected SPN condition estimate was observed at Cz, but this effect did not survive FDR correction. In addition, the supplementary ROI-interaction model did not show reliable Condition × ROI effects, and the exploratory cluster-based permutation analyses did not identify suprathreshold spatial clusters for the *in noise* minus *in silence* contrast in either SPN or CNV. Therefore, the central tendency should be interpreted as a descriptive feature of the present data rather than as evidence for a reliable condition-specific central SPN enhancement.

The response-locked CNV control analysis further supported this interpretation. If the effect of noise on the SPN reflects a global increase in the preparatory or attentional state, a similar condition effect may also have been expected in CNV. However, CNV analysis showed no reliable main effect of the condition. This convergence across the SPN and CNV suggests that, in the present dataset, anticipatory slow potentials were present, but were not systematically modulated by the listening context. Accordingly, the current results do not support the conclusion that noisy listening robustly heightens feedback- or response-locked anticipatory negativity. The supplementary Bayes factor analyses favored reduced models without condition-related terms for both SPN and CNV. This pattern is consistent with the frequentist LMMs in suggesting that the present data did not strongly support condition-related modulation. However, these Bayes factors were based on BIC approximation and depend on model specification. Therefore, they should be interpreted as sensitivity analyses rather than as definitive evidence that condition effects are absent. The independent speech-in-noise test showed that recognition accuracy declined as the SNR became poorer, indicating that the behavioral test captured the expected effect of increasing acoustic difficulty across SNR levels. However, this result should be distinguished from the EEG manipulation, which contrasted silence with 0 dB SNR noise. Because performance at 0 dB SNR was high and interindividual variability was limited in this young normal-hearing sample, the noise condition during EEG recording may not have imposed sufficient perceptual difficulty to produce a reliable increase in anticipatory slow potentials. The broadly similar feedback-based task success across listening conditions is also consistent with the possibility that the noise condition did not introduce a large cognitive disturbance during the time-estimation task. However, because absolute timing error was not analyzed separately, the feedback-based measure should be interpreted as an indirect index of task success rather than as a detailed measure of timing precision. Under these circumstances, it is perhaps unsurprising that exploratory brain–behavior analyses did not reveal any robust associations between speech-in-noise accuracy and either SPN or CNV measures. Therefore, the present data do not support the conclusion that larger anticipatory slow potentials are directly linked to better speech-in-noise performance in this paradigm.

These findings appear to be weaker than those reported in our prior study, which suggested larger SPN amplitudes under background noise (Okamoto et al., 2024). Thus, we view the present results not as a direct contradiction, but as a more conservative re-evaluation of our prior studies in an independent cohort using a revised analysis pipeline. In particular, the present study prioritized non-interpolated primary analyses, reported exploratory spatial analyses without using them to define confirmatory follow-up ROIs, and treated brain-behavioral analyses as exploratory. Under this stricter framework, the evidence for a reliable listening condition effect is reduced. Accordingly, the present study indicates that any enhancement of anticipatory negativity by background noise is likely to be smaller and less robust than implied by our earlier findings.

This study had some limitations. First, a 0.5 Hz high-pass filter was applied during acquisition. This preprocessing attenuates slow potentials, such as the SPN, and may distort their morphology; therefore, the reported amplitudes should be regarded as conservative estimates (Tanner et al., 2015). Nevertheless, the relative condition differences of the SPN remain interpretable, as these features are less sensitive to the overall amplitude scaling.

Second, the final EEG sample included 20 participants, which may have limited sensitivity to small condition effects or individual differences in condition-related modulation. Although the supplementary Bayes factor analyses favored reduced models without condition-related terms, these results should be interpreted alongside the modest sample size and the present task parameters. Therefore, the absence of statistically reliable condition effects should not be interpreted as evidence that background noise cannot modulate anticipatory slow potentials. Rather, the present results indicate that such modulation was not robustly detected under the present sample characteristics and experimental conditions.

Third, the sample comprised only young adults with self-reported normal hearing, which may have restricted inter-individual variability in speech-in-noise performance, particularly at easier SNR levels. This limitation is particularly relevant because the EEG noise condition used 0 dB SNR, at which recognition accuracy was close to ceiling in the present sample. Previous research has shown that older adults rely more on predictive processes during perception, likely because of reduced sensory precision and accumulated perceptual experience (Chan et al., 2021, Henry et al., 2017, Kocagoncu et al., 2021). Moreover, the age-related attenuation of neural responses to prediction errors has been reported (Bornkessel-Schlesewsky et al., 2015, Trapp et al., 2022). These findings indicate that the results may not be generalizable to older individuals or those with hearing loss. Future studies should therefore include participants of varying ages and hearing profiles to examine the broader applicability of these findings.

Fourth, the EEG was recorded using a low-density 19-channel montage. This limited spatial resolution motivated the use of a conservative non-interpolated primary analysis. Although this choice reduced the risk of overinterpreting the interpolated signals, it may have reduced the sensitivity to weak condition effects. At the same time, supplementary sensitivity analyses comparing non-interpolated and interpolated models yielded the same overall pattern of results for both SPN and CNV, suggesting that the main conclusions were not driven by the decision to avoid interpolation in the primary analyses.

Fifth, we elicited SPN using a time-estimation paradigm with auditory feedback, which likely conflates temporal prediction with content-based prediction (the anticipation of feedback correctness). In this task, participants may have formed not only content-based predictions based on the perceived accuracy of their responses, but also temporal predictions about the timing of the feedback stimulus. Because temporal and content-based predictions may engage partially distinct neural mechanisms (Auksztulewicz et al., 2018), the present paradigm makes it difficult to determine the type of prediction that the SPN primarily reflects. Future research should orthogonally manipulate SOA uncertainty, lexical probability, and context to isolate these components. Incorporating variable SOAs or an explicit hazard function to manipulate the trial-wise temporal uncertainty would allow a more direct test of the relationship between task-specific temporal prediction and SPN. Broadly, this interpretive ambiguity is not limited to the distinction between temporal and content-based predictions. Furthermore, the present design does not allow any prediction to be clearly separated from the attentional allocation, listening effort, or other preparatory processes. Under noisy listening conditions, listeners may rely more on contextual or expectancy-related information. However, noise may further increase their attention and effort. Accordingly, any condition-related variation in anticipatory slow potentials should be interpreted cautiously, as it cannot be specifically attributed to predictive processing in the present paradigm.

In conclusion, the present study showed that the time estimation task elicited a clear posteriorly distributed anticipatory slow potential prior to auditory feedback. However, in this independent cohort, and under the conservative non-interpolated analytical framework, neither the SPN nor the response-locked CNV showed any reliable modulation by listening conditions. Speech-in-noise performance further exhibited the expected dependence on SNR, but did not show any robust associations with the anticipatory EEG measures. These results indicate that caution is warranted when interpreting anticipatory slow potentials under noisy listening conditions. Specifically, slow potentials seem to reflect the anticipatory neural activity elicited by the task; however, their modulation by background noise and their relationship with behavioral performance should be interpreted cautiously. These findings should not be interpreted as evidence that background noise cannot modulate anticipatory slow potentials. Rather, any such modulation was not robustly detected under the present conditions, including the modest final sample size, young normal-hearing participants, and near-ceiling recognition accuracy at 0 dB SNR.

## **Compliance with Ethical Standards**

This study was approved by the Research Ethics Committee of Fukui University of Health Sciences (Approval No. 2022–14) and conducted in accordance with the Declaration of Helsinki. The cohort included 26 participants (13 females, 13 males), all of whom provided written informed consent before enrollment.

## **Funding**

This study was supported by a Grant-in-Aid for Scientific Research from the 10.13039/501100001691Japan Society for the Promotion of Science (KAKENHI 22K17620). The funder played no role in the study design, data collection, analysis, or reporting.

## 7 Data Statement

De-identified raw EEG data, derived analysis tables, participant metadata, and quality-control documentation are publicly available at https://doi.org/10.5281/zenodo.19344047. The shared dataset includes raw EEG recordings together with analysis-ready non-interpolated derivatives for the revised primary analyses, supplementary interpolated derivatives, and accompanying documentation describing participant- and block-level exclusions. The complete analysis code is available at https://github.com/kokamoto46/analyze_spn.

## CRediT authorship contribution statement

**Yasutaka Kobayashi:** Writing – review & editing, Supervision, Resources, Methodology. **Narihiro Kodama:** Writing – review & editing, Methodology. **Tomomi Nomura:** Writing – review & editing, Formal analysis. **Kengo Hoyano:** Writing – review & editing, Methodology, Conceptualization. **Naoya Obama:** Writing – review & editing, Formal analysis. **Keisuke Irie:** Writing – review & editing, Methodology. **Kazuhiro Okamoto:** Writing – review & editing, Writing – original draft, Visualization, Resources, Project administration, Methodology, Investigation, Funding acquisition, Formal analysis, Data curation, Conceptualization.

## Declaration of Competing Interest

None.
