# Supplementary material for: Anticipatory slow potentials before auditory feedback show posterior predominance but limited condition effects in speech-in-noise
Source: IBRO Neurosci Rep. 2026 Jun 1;21:32–41. doi: 10.1016/j.ibneur.2026.05.013 (PMC13254385; doi:10.1016/j.ibneur.2026.05.013)
Supplement: Table S1 — Supplementary material [file mmc1.pdf]

Supplementary Tables

This file contains six supplementary tables accompanying the manuscript:

Table S1, electrode-wise linear mixed-effects models for SPN amplitude with false discovery rate correction;

Table S2, sensitivity analysis comparing non-interpolated and interpolated ROI × hemisphere models for SPN and CNV;

Table S3, electrode-wise linear mixed-effects models for CNV amplitude with false discovery rate correction;

Table S4, exploratory participant-level associations between speech-in-noise performance and anticipatory EEG measures;

Table S5, Supplementary Condition × ROI linear mixed-effects models for SPN and CNV amplitudes;

Table S6, Supplementary BIC-approximated Bayes factor analyses for condition-related terms in SPN and CNV models.

Table S1. Electrode-wise linear mixed-effects models for SPN amplitude with false discovery rate correction

ROI	Electrode	*b* (µV)	SE (µV)	*p*	*q* (FDR)
**Frontal pole**	Fp1	0.191	0.224	0.392	0.680
	Fp2	0.423	0.260	0.103	0.620
**Frontal**	F7	-0.335	0.305	0.271	0.630
	F3	0.167	0.368	0.649	0.863
	Fz	0.489	0.357	0.172	0.620
	F4	0.373	0.345	0.280	0.630
	F8	-0.210	0.258	0.415	0.680
**Central**	T3	NA	NA	NA	NA
	C3	0.482	0.364	0.185	0.620
	Cz	0.740	0.394	0.061	0.620
	C4	0.461	0.365	0.207	0.620
	T4	-0.209	0.706	0.767	0.863
**Parietal**	T5	-0.209	0.706	0.767	0.863
	P3	0.657	0.382	0.086	0.620
	Pz	0.350	0.469	0.456	0.684
	P4	-0.016	0.440	0.970	0.970
	T6	0.059	0.378	0.877	0.928
**Occipital**	O1	0.160	0.407	0.694	0.863
	O2	0.300	0.355	0.397	0.680

Abbreviations: SPN = stimulus-preceding negativity; *b* (µV) = fixed-effect coefficient (Condition main effect); SE (µV) = standard error of *b*; *p* = two-sided uncorrected *p*-value; *q* (FDR) = Benjamini–Hochberg false-discovery-rate–adjusted *p*-value; µV = microvolts. **Note:** Electrode-wise supplementary analyses tested the main effect of Condition on non-interpolated SPN amplitude. *P* values were adjusted using the Benjamini–Hochberg false discovery rate procedure. NA indicates that the model could not be estimated for that electrode because of insufficient usable data after quality-control filtering.

Table S2. Sensitivity analysis comparing non-interpolated and interpolated ROI × hemisphere models for SPN and CNV amplitudes

(A) Stimulus-preceding negativity

	**Non-interpolated *b* (SE)**	*z*	*p*	**Interpolated *b* (SE)**	*z*	*p*
ROI: Frontal	0.333 (0.118)	2.823	0.005	0.288 (0.102)	2.816	0.005
ROI: Occipital	−0.730 (0.129)	-5.662	< 0.001	−0.745 (0.114)	-6.525	< 0.001
ROI: Parietal	−0.600 (0.116)	-5.181	< 0.001	−0.599 (0.102)	-5.864	< 0.001
Condition	0.318 (0.361)	0.878	0.380	0.381 (0.322)	1.183	0.237
Hemisphere	0.046 (0.100)	0.459	0.647	0.047 (0.088)	0.529	0.597
Condition × Hemisphere	0.139 (0.199)	0.700	0.484	0.191 (0.177)	1.081	0.280

(B) Contingent negative variation

	**Non-interpolated *b* (SE)**	*z*	*p*	**Interpolated *b* (SE)**	*z*	*p*
ROI: Frontal	0.318 (0.114)	2.780	0.005	0.191 (0.099)	1.927	0.054
ROI: Occipital	−0.006 (0.125)	-0.050	0.960	−0.075 (0.111)	-0.676	0.499
ROI: Parietal	−0.134 (0.112)	-1.198	0.231	−0.188 (0.099)	-1.891	0.059
Condition	−0.058 (0.361)	-0.158	0.874	−0.099 (0.336)	-0.296	0.767
Hemisphere	0.111 (0.096)	1.154	0.249	0.066 (0.086)	0.766	0.444
Condition × Hemisphere	0.033 (0.193)	0.170	0.865	0.080 (0.172)	0.465	0.642

Abbreviations: *b* = fixed-effect coefficient; SE = standard error; *z* = Wald z statistic; *p* = two-sided p-value; ROI = region of interest. Values are fixed-effect estimates from supplementary sensitivity analyses comparing the primary non-interpolated models with corresponding interpolated models. Intercepts are omitted for brevity.

Table S3. Electrode-wise linear mixed-effects models for CNV amplitude with false discovery rate correction

ROI	Electrode	*b* (µV)	SE (µV)	*p*	*q* (FDR)
**Frontal pole**	Fp1	-0.379	0.272	0.163	0.906
	Fp2	-0.100	0.315	0.751	0.906
**Frontal**	F7	-0.282	0.287	0.326	0.906
	F3	-0.507	0.387	0.190	0.906
	Fz	-0.438	0.425	0.303	0.906
	F4	-0.488	0.395	0.216	0.906
	F8	-0.153	0.232	0.508	0.906
**Central**	T3	NA	NA	NA	NA
	C3	-0.177	0.375	0.637	0.906
	Cz	-0.146	0.437	0.739	0.906
	C4	0.277	0.527	0.599	0.906
	T4	0.201	0.743	0.787	0.906
**Parietal**	T5	0.285	0.296	0.336	0.906
	P3	0.067	0.400	0.868	0.916
	Pz	0.184	0.447	0.680	0.906
	P4	0.147	0.393	0.708	0.906
	T6	0.093	0.339	0.784	0.906
**Occipital**	O1	0.376	0.392	0.338	0.906
	O2	0.077	0.320	0.810	0.906

Abbreviations: CNV = contingent negative variation; *b* (µV) = fixed-effect coefficient (Condition main effect); SE (µV) = standard error of *b*; *p* = two-sided uncorrected *p*-value; *q* (FDR) = Benjamini–Hochberg false-discovery-rate–adjusted *p*-value; µV = microvolts. **Note:** Electrode-wise supplementary analyses tested the main effect of Condition on non-interpolated response-locked CNV amplitude. *P* values were adjusted using the Benjamini–Hochberg false discovery rate procedure. NA indicates that the model could not be estimated for that electrode because of insufficient usable data after quality-control filtering.

Table S4. Exploratory participant-level associations between speech-in-noise performance and anticipatory EEG measures

Speech measure	EEG measure	N	Pearson *r*	Pearson *p*	Spearman *ρ*	Spearman *p*
Accuracy at 0 dB	SPN (noise - silence)	20	-0.191	0.419	-0.061	0.798
Accuracy at 0 dB	SPN in noise	20	-0.114	0.632	-0.044	0.855
Accuracy at 0 dB	CNV (noise - silence)	20	-0.138	0.56	0.27	0.249
Accuracy at 0 dB	CNV in noise	20	-0.263	0.262	-0.305	0.191
Accuracy across all SNRs	SPN (noise - silence)	20	-0.145	0.542	-0.067	0.779
Accuracy across all SNRs	SPN in noise	20	-0.325	0.162	-0.403	0.078
Accuracy across all SNRs	CNV (noise - silence)	20	-0.218	0.356	0.052	0.826
Accuracy across all SNRs	CNV in noise	20	-0.341	0.141	-0.379	0.099
Accuracy at difficult SNRs	SPN (noise - silence)	20	-0.09	0.706	-0.082	0.73
Accuracy at difficult SNRs	SPN in noise	20	-0.302	0.196	-0.346	0.135
Accuracy at difficult SNRs	CNV (noise - silence)	20	-0.183	0.441	-0.153	0.519
Accuracy at difficult SNRs	CNV in noise	20	-0.271	0.248	-0.296	0.205

Abbreviations: SPN = stimulus-preceding negativity; CNV = contingent negative variation; SNR = signal-to-noise ratio. "noise - silence" indicates the participant-level difference between the *in noise* and *in silence* conditions. "Difficult SNRs" refers to the mean accuracy across -5, -10, and -15 dB SNR. Pearson *r* and Spearman *ρ* denote Pearson's and Spearman's correlation coefficients, respectively. **Note:** These analyses were exploratory and were not treated as primary inferential tests.

Table S5. Supplementary Condition × ROI linear mixed-effects models for SPN and CNV amplitudes.

(A) Stimulus-preceding negativity

	b **(µV)**	**SE**	***z***	***p***	**95% CI**
ROI: Frontal	0.335	0.118	2.838	0.005	[0.104, 0.566]
ROI: Occipital	-0.728	0.129	-5.648	< 0.001	[-0.981, -0.476]
ROI: Parietal	-0.598	0.116	-5.168	< 0.001	[-0.825, -0.371]
Condition × ROI: Frontal	-0.225	0.236	-0.952	0.341	[-0.687, 0.238]
Condition × ROI: Occipital	-0.297	0.258	-1.151	0.250	[-0.802, 0.209]
Condition × ROI: Parietal	-0.249	0.232	-1.074	0.283	[-0.702, 0.205]
Condition	0.512	0.393	1.302	0.193	[-0.259, 1.282]
Hemisphere	0.047	0.100	0.471	0.638	[-0.148, 0.242]
Condition × Hemisphere	0.124	0.199	0.625	0.532	[-0.266, 0.515]

(B) Contingent negative variation

	b **(µV)**	**SE**	***z***	***p***	**95% CI**
ROI: Frontal	0.319	0.114	2.791	0.005	[0.095, 0.543]
ROI: Occipital	-0.008	0.125	-0.061	0.951	[-0.253, 0.237]
ROI: Parietal	-0.135	0.112	-1.206	0.228	[-0.355, 0.085]
Condition × ROI: Frontal	-0.251	0.228	-1.097	0.273	[-0.698, 0.197]
Condition × ROI: Occipital	0.368	0.250	1.476	0.140	[-0.121, 0.857]
Condition × ROI: Parietal	0.317	0.224	1.418	0.156	[-0.121, 0.756]
Condition	-0.155	0.389	-0.398	0.691	[-0.917, 0.608]
Hemisphere	0.112	0.096	1.162	0.245	[-0.077, 0.301]
Condition × Hemisphere	0.041	0.193	0.213	0.831	[-0.337, 0.419]

Abbreviations: *b* = fixed-effect coefficient; SE = standard error; *z* = Wald z statistic; *p* = two-sided p-value; 95% CI = 95% confidence interval; SPN = stimulus-preceding negativity; ROI = region of interest; µV = microvolts. **Note:** The central ROI served as the reference level for the ROI factor. Therefore, the Condition term represents the estimated condition effect at the central ROI, and each Condition × ROI term represents the difference in the condition effect between the corresponding ROI and the central ROI. Condition was sum-coded as *in noise* = -0.5 and *in silence* = +0.5. Positive condition coefficients indicate relatively more positive amplitudes in silence than in noise, corresponding to relatively greater negativity in noise.

Table S6. Supplementary BIC-approximated Bayes factor analyses for condition-related terms in SPN and CNV models.

Component	Comparison	BIC reduced	BIC full	ΔBIC	BF01
SPN	Condition main effect only	143349.64	143359.45	9.81	134.94
SPN	All condition-related terms in primary ROI × Hemisphere model	143349.64	143368.95	19.31	15571.05
SPN	Added Condition × ROI terms beyond primary model	143368.95	143397.28	28.33	1418802.22
CNV	Condition main effect only	142729.19	142739.19	10.00	148.39
CNV	All condition-related terms in primary ROI × Hemisphere model	142729.19	142749.16	19.98	21786.42
CNV	Added Condition × ROI terms beyond primary model	142749.16	142769.47	20.31	25668.70

Abbreviations: SPN = stimulus-preceding negativity; CNV = contingent negative variation; ROI = region of interest; BIC = Bayesian information criterion; ΔBIC = BIC full – BIC reduced; BF01 = approximate Bayes factor supporting the reduced model over the full model. **Note:** Bayes factors were approximated from BIC differences using maximum-likelihood model fits. For the Condition main-effect comparison, the reduced model excluded Condition and the full model included Condition. For the condition-related-terms comparison, the full model included Condition and Condition × Hemisphere terms. For the Condition × ROI comparison, the reduced model was the primary ROI × hemisphere model and the full model additionally included Condition × ROI terms. These analyses were conducted as supplementary sensitivity analyses and should not be interpreted as fully Bayesian mixed-effects models with explicit prior distributions.
